# Impact of Freeze-thaw Cycles and Storage Time on Plasma Samples Used in Mass Spectrometry Based Biomarker Discovery Projects

**Published:** 2007-02-22

**Authors:** Breeana L Mitchell, Yutaka Yasui, Christopher I Li, Annette L. Fitzpatrick, Paul D Lampe

**Affiliations:** a Public Health Sciences,; b Fred Hutchinson Cancer Research Center, Molecular and Cellular Biology Program,; c Departments of Epidemiology, and; d Pathobiology, University of Washington; e Department of Public Health Sciences University of Alberta

**Keywords:** mass spectrometry, proteomics, long-term storage, biomarker, plasma

## Abstract

Mass spectrometry approaches to biomarker discovery in human fluids have received a great deal of attention in recent years. While mass spectrometry instrumentation and analysis approaches have been widely investigated, little attention has been paid to how sample handling can impact the plasma proteome and therefore influence biomarker discovery. We have investigated the effects of two main aspects of sample handling on MALDI-TOF data: repeated freeze-thaw cycles and the effects of long-term storage of plasma at −70°C. Repeated freeze-thaw cycles resulted in a trend towards increasing changes in peak intensity, particularly after two thaws. However, a 4-year difference in long-term storage appears to have minimal effect on protein in plasma as no differences in peak number, mass distribution, or coefficient of variation were found between samples. Therefore, limiting freeze/thaw cycles seems more important to maintaining the integrity of the plasma proteome than degradation caused by long-term storage at −70°C.

## Introduction

Biomarkers have the potential to aid in the diagnosis and treatment of many diseases, from cancer to heart disease. The advent of new technologies has generated increased interest in widespread screening for disease biomarkers. Identifying biomarkers derived from human serum or plasma is particularly appealing because blood can be obtained with high patient acceptability and it is in contact with most tissues, thereby making it likely to contain proteins or peptides that could be used to distinguish between individuals with or without a particular disease.

Recent advances in mass spectrometry technologies have increased the accuracy and sensitivity of this tool such that it can now be practically used for the analysis of complex mixtures such as blood. Matrix based time-of-flight mass spectrometry techniques have been pursued extensively in recent years due to high sample throughput and ease of use. Matrix assisted laser desorption/ionization time-of-flight (MALDI-TOF) and surface enhanced laser desorption/ionization time-of-flight (SELDI-TOF) are similar methods based on the laser excitation and subsequent unique flight time of proteins or peptides in a complex mixture (Hillenkamp and Karas 1990; Hutchens 1993). Recent publications using primarily SELDI-TOF have reported the ability to classify certain diseases with extremely high sensitivity and specificity (Adam et al. 2002; Petricoin et al. 2002b; Petricoin et al. 2002a; Banez et al. 2003; Won et al. 2003; Alexe et al. 2004). Despite the seeming success of early studies, mass spectrometry TOF-based biomarker discovery is fraught with controversy (Diamandis 2002; Diamandis 2003; Sorace and Zhan 2003; Baggerly et al. 2004; Diamandis 2004). From the first stages of sample collection to the final stages of data analysis, the methods and techniques employed are likely to have considerable impact on the outcome of a study.

While careful consideration is often paid to the development of the mass spectrometry protocol and analysis of mass spectrometry data, most studies have not considered how the handling of the samples prior to mass spectrometry analysis could affect the study results. One appeal of using serum or plasma for biomarker discovery projects is that there are many excellent epidemiological studies that have already been conducted. Some of these studies have obtained blood from participants and a few have sera or plasma samples available from multiple time points, including samples drawn prior to disease diagnosis. These samples could contain a wealth of information that was inaccessible before the advances in mass spectrometry. However, a caveat of biomarker discovery using mass spectrometry is that the technique relies on the consistency of the mass of proteins and peptides between samples and over time. Improper sample storage may lead to protein degradation, reducing the power of a study to detect differences within a population. As of yet, we are not aware of any studies investigating the effects that sample storage and handling could have on the outcome of biomarker discovery projects using mass spectrometry.

In this study, we address two aspects of sample storage. We used control plasma to investigate whether repeated freeze-thaw cycles have an effect on the quality of a plasma sample. Further, using samples obtained at two time points four years apart in a cohort study, we investigated the significance of long-term storage of plasma samples at −70°C.

## Methods

### Longitudinal Study participants

This study utilizes blood samples obtained from participants of the Cardiovascular Health Study (CHS), an observational cohort sponsored by the National Heart, Lung and Blood Institute to investigate risk factors for cardiovascular disease in the elderly (Fried et al. 1991). The CHS multi-site cohort includes 5,201 men and women aged 65 or older who were enrolled in 1989/90 and an additional 687 African-Americans enrolled in 1992/93. Informed consent was obtained from all study subjects. Eligibility for the CHS study was limited to those 65 or older who were noninstitutionalized and were expected to reside in the study area for the next three years. Subjects were excluded if they were wheelchair-bound, receiving hospice care, or had received radiation or chemotherapy for cancer prior to enrollment (Tell et al. 1993). For this sub-study, we obtained frozen plasma samples from 50 randomly selected CHS healthy subjects (dementia, cancer, and cardiovascular disease-free through 1996/97) who had visited the CHS clinic for his/her annual examination in 1992/93 (year 5 of the study) and 1996/97 (year 9 of the study). For each collection, blood was drawn in the morning following an 8–12 hour fast and collected into EDTA-containing tubes. Immediately following collection, blood was mixed at 4°C and spun at 3,000g for 10min at 4°C. Plasma samples were then frozen at −70°C and shipped on dry ice to the storage facility in the department of Pathology/Biochemistry/Medicine at the University of Vermont (Fried et al. 1991; Cushman et al. 1995).

The mean age of the 50 subjects included in this study was 74.4 years (+/− 3.4 years) (Table 1). Thirty-three subjects were female and 41 were white. Samples from all four CHS clinical sites were included.

### Acetonitrile precipitation

Plasma samples were thawed and proteins were precipitated from a 20μl aliquot by the addition of an equal volume of acetonitrile. Acetonitrile treated samples were mixed at room temperature for 30min using a multi-speed vortexer at the lowest setting. Precipitated proteins were pelleted by centrifugation at 12,000rpm for 4min at room temperature; the supernatant was removed and stored on ice for analysis.

### MALDI-TOF

A 384 well hydrophobic MALDI-TOF plate was pre-crystallized with sinapinic acid matrix, a supersaturated solution of 4-hydroxy-3,5 dimethoxycinnamic acid in acetonitrile/0.1% TFA (50/50 v/v). Acetonitrile treated plasma samples were mixed with sinapinic acid in a 1:3 ratio. Each sample was spotted in quintuplicate onto the pre-crystallized plate. Mass accuracy was obtained by daily machine calibrations using cytochrome c [12361m/z (+1), 6181m/z (+2)]. The plate was analyzed in an Applied Biosystems Voyager mass spectrometer in linear mode, using a 337 nm nitrogen laser. Settings were optimized using cytochrome c and were: laser intensity 1784, accelerating voltage 25000, grid voltage 23000, guide wire voltage 1250, delay time 325ns, low mass gate 1000, molecular mass range 1000 – 40,000. Final spectra were generated by summing spectra produced from 20 laser hits at up to 20 places per spot (a total of up to 400 laser hits per summed spectra). Individual spectra had to have an intensity of 3000 hits or more in order to be used in the summed spectra. The operator was blinded to the identity of the samples.

### Freeze-thaw experiment

Three plasma samples from the same person were obtained. The samples were thawed at room temperature. One aliquot of 20μl was taken from each sample (freeze-thaw 1). Samples were re-frozen at −70°C for one hour. The original samples were thawed again at room temperature and a second aliquot of 20μl was taken from each sample (freezethaw 2). Samples were re-frozen at −70°C for one hour. This cycle was repeated five times to yield five freeze-thaw samples. All samples were then precipitated at the same time with an equal volume of acetonitrile and run in quintuplicate on the MALDITOF instrument.

### Data Analysis

Multiple methods for defining and aligning MALDITOF MS spectrum have been devised and this is an active area of research but is not the focus of this report. Briefly, our method defined “potential peaks” by judging, at each mass/charge point, whether or not the protein intensity at that point is the highest among its nearest ± 10 points in the mass/chargeaxis direction. If it was the highest, that point was defined as a potential peak. The number of points defined as a peak is arbitrary and will vary with the width of the “window” used to define a peak. See Yasui et al for a discussion of how the ± 10 points window was chosen and a detailed description of the method (Yasui et al. 2003). We then estimated the noise level in the neighborhood of each point by a successive application of smoothing techniques and assessed whether the intensity at each potential peak is at least 3 times higher than the noise level in its neighborhood. Those potential peaks that satisfied this 3-fold-intensity criterion were defined as peaks. Peak alignment was performed by considering a window of ±0.1% of the mid mass/charge value, within which all peaks are considered to represent an identical peak: ±0.1% of mass/charge value was selected based on the mass/charge-axis shifting estimated from a QC experiment. We successively identified the window that contained the highest number of peaks among all possible windows and, for each spectrum, assigned the mid-mass/charge value of the window, coupled with the maximum intensity value of the spectrum within the window, as its (aligned mass/charge, intensity) pair. This procedure was repeated until every peak was assigned an aligned mass/charge value. The end product of this repeated procedure is an aligned data matrix that consists of (aligned mass/charge, intensity) pairs of all spectra. The aligned spectra were log transformed to facilitate linear modeling and the aligned data matrix were normalized by subtracting the spectrum-specific mean so that the normalized log intensity values of every spectrum has zero mean.

Following the peak identification/alignment and intensity normalization, we performed a variance-component analysis using the following linear mixed model:

$$Y_{ijk}^{\left(m\right)}=\mu_{5}^{\left(m\right)}+\delta_{59}^{\left(m\right)}+b_{5i}^{\left(m\right)}+b_{9_{i}}^{\left(m\right)}{+}_{ijk}^{\left(m\right)}$$

where

$$Y_{ijk}^{\left(m\right)}$$

is the normalized log-intensity measurement of mth peak for *i*^th^ subject’s *j*^th^ time’s *k*^th^ replicate,

$$\mu_{5}^{\left(m\right)}$$

is the overall mean log-intensity of *m*^th^ peak in 1992/93,

$$\delta_{59}^{\left(m\right)}$$

is the mean log-intensity difference of *m*^th^ peak from 1992/93 to 1996/97,

$$\left(b_{5i}^{\left(m\right)}\;\;\;b_{9i}^{\left(m\right)}\right)$$

are a bivariate mean-zero Gaussian random effect of *m*^th^ peak for *i*^th^ subject with the variance-covariance matrix:

$$\left[\begin{array}{cc} \sigma_{5}^{(m)2} & {p_{5}^{\left(m\right)}}_{9}^{\left(m\right)}\\ p\sigma_{5}^{\left(m\right)}\sigma_{9}^{\left(m\right)} & \sigma_{9}^{{\left(m\right)}^{2}} \end{array}\right]\;\;\;\sigma$$

representing the individual-specific fluctuations from the means, and

$${ɛ}_{ijk}^{\left(m\right)}$$

is the independent mean-zero Gaussian error term with variance

$$\tau^{(m)2}$$

Restricted maximum-likelihood estimates were obtained for the model parameters.

The reliability of the MALDI-TOF MS intensity measurements was assessed by computing coefficients of variations (CV) for each peak (log scale) in each time point. For the 1992/93 samples, the CV of *m*^th^ peak was given by

$$CV_{5}^{\left(m\right)}={\left(\sigma_{5}^{{\left(m\right)}^{2}}+\tau^{(m)2}\right)}^{1/2}{/}_{5}^{\left(m\right)}$$

For the 1996/97 samples, the variance and mean parameter estimates in the formula were replaced with those of 1996/97. Inference on the parameter provided the within-subject correlation between ρ provided the within-subject correlation between 1992/93 and 1996/97 log-intensity measurements.

The analysis of the freeze-thaw experiment followed the same peak identification/alignment and intensity normalization procedures.

## Results

### Freeze-Thaw effects on MALDI Signal Intensity

We investigated the effect of repeated freeze-thaw cycles on peak intensity with each cycle. The change in peak intensity is reported as an absolute percentage value because we observed both positive and negative changes in peaks of wide ranging intensities (Table 2). The second, third, fourth and fifth freeze-thaw cycles changed a given peak intensity by a median of 1.7%, 2.4%, 3.5% and 3.1% respectively, compared to the intensity measurements of the sample freeze-thawed once. While this indicates that the proteins are generally relatively stable, the range of intensity change was quite high. For example 1% of the proteins changed by at least 67% after 4 freeze/thaw cycles (Table 2, 99^th^ percentile change column). We also investigated the changes in peak intensity following freeze-thaw cycles by mass/charge range (Table 3). Peaks at ≥20,000m/z and 10,000–20,000m/z had a reduction in median peak intensity while at the 5,000–10,000m/z range there was in increase in peak intensity.

### Effects of Long Term Storage at −70°C

There were 2,153 aligned mass/charge values at which peaks were identified in the 500 MALDI spectra (50 subjects × 2 samples collected 4 years apart per subject × 5 replicates per sample). The number of peaks per spectrum in the 1992/93 sample spectra ranged from 545 to 938 (median=636, 25^th^-percentile=611, 75^th^-percentile=666), and from 552 to 897 (median=630, 25^th^-percentile=596, 75^th^ percentile=673) in the 1996/97 sample spectra (Table 4). These numbers of peaks per spectrum are consistent with our routine analysis of plasma samples. When using only a single MALDI run for each sample, the median CV for the 2,153 peaks in the 1992/93 samples was 14.6% (25^th^-percentile=11.6%, 75^th^-percentile=26.3%) and was 13.7% (25^th^ percentile=11.3%, 75^th^-percentile=26.5%) in the 1996/97 samples. Using the 5 replicates reduced the median CVs of peaks from 14.6% to 11.1% for the 1992/93 samples, and from 13.7% to 10.2% for the 1996/97 samples (Table 4). To calculate the correlation of intensity measures between the 1992/93 and the 1996/97 samples, we estimated the correlation parameter using log-intensity measures. The correlations ranged from −0.14 to 0.95, with a median of 0.40 and 25^th^- and 75^th^-percentiles of 0.32 and 0.47, respectively (Table 4). When the number of peaks was compared at different mass values there was no appreciable difference between the 1992/93 samples and the 1996/97 samples at ranges of <5,000mass/charge, 5,000 to 10,000mass/charge and >10,000mass/charge (Table 5).

## Discussion

Peak intensity appears to be influenced by repeated freeze-thawing of samples. While the change in median peak intensity is small, the impact on the data could be significant if samples are not consistently treated and/or if samples are not carefully tracked for freeze-thaw status. Some proteins or peptides are more sensitive to freeze-thaw cycles than others. This is illustrated by the high percentage of intensity changes seen in the 95^th^ and 99^th^ percentile of peaks (Table 2). Further, analysis of the change in peak intensity by mass range shows that on average, higher mass peaks lose intensity while peaks in the smaller mass ranges show modestly increased peak intensity as would be expected if the larger proteins were more susceptible to degradation. Based on these results, freeze-thawing a sample more than twice could begin to compromise data quality by interfering with peak detection. Differences in peak intensity between disease states may not be evident if peaks are significantly degraded by multiple freeze-thaw exposures. Further, these results indicate that it would be inadvisable to use control samples that had been freeze-thawed a different number of times than the cases (i.e., controls from a different study) as particularly sensitive peaks might track with freeze-thaw treatment rather than disease status. To our knowledge, this is the first study to report how repeated freeze-thaw cycles can affect plasma mass spectrometry data quality.

We also investigated how long-term storage at −70°C influenced MADLI mass spectrometry data using samples that were frozen in 1992/1993 compared to samples frozen 4 years later. While it is possible that there may have been some protein degradation that has occurred in the five years since the 1996/97 samples were frozen, samples stored in a similar manner represent some of the best samples available for biomarker discovery work. Thus, we feel that these samples correspond to a time frame that is representative of the samples that we, and others, are using for disease marker identification.

The MALDI measurements in the range of 1,000 to 40,000m/z of the 1992/93 plasma samples showed no difference in the number of peaks compared to the 1996/97 samples that were collected four years more recently. Comparing the number of peaks by mass ranges also reveals no differences between the two sample years. If long-term storage at −70°C caused significant protein degradation, one would expect to see more small peaks in the 1992/93 samples than in the newer, 1996/97 samples. However, this is not the case, suggesting that the length of freezer storage at −70°C in a study will not affect the quality of MALDI assays. Further, using all five replicates, the MALDI measurements of the 1992/93 plasma samples had a median CV of 11.1%, which does not differ appreciably from the median CV of 10.2% for the 1996/97 plasma samples. This indicates that well over 1,000 peaks (of the total of 2,153 identified) were measured with a CV of <15%. As described above, this level of CV is definitely acceptable and compares well with many laboratory assays used in biomedical research.

The within-subject correlation of the two MALDI measurements taken 4 years apart was moderate (a median correlation of 0.40). This is consistent with our expectation that the longitudinal protein profile of a subject will remain stable to some degree, but also is consistent with the fact that the proteome is dynamic and is influenced by a variety of factors including physiological changes (e.g., the aging process), diet, and transient exposures, all of which could vary appreciably over 4 years, particularly in this aging population. Further, while the samples were randomized and run on the same plate on the same day, instrument variation could also reduce the correlation between the samples.

Consistent with our results, a previous study found no change in nine risk factors for anthero-sclerosis and thrombosis in plasma pools that had been stored at −70°C for various lengths of time (Lewis et al. 2001). While the study by Lewis et al used functional or immunoassays to measure retention of epitopes over time, by taking advantage of the properties of mass spectrometry we were able to evaluate long-term storage effects on the protein stability of plasma samples. This is the first study, to our knowledge, to report on the effects of long-term storage on a broad screen of the human plasma proteome.

In conclusion, it is evident from our freeze-thaw experimental data that repeatedly thawing samples can lead to protein degradation. In fact, repeated thawing appears to be more problematic than long-term storage at −70°C in terms of maintenance of peak number and intensity. This finding is encouraging because the number of freeze-thaw cycles a sample is subjected to can be monitored and controlled by early sample aliquoting whereas the length of sample storage is often not something that can be changed. The use of plasma samples from longitudinal studies may be an ideal approach to biomarker discovery as they often allow a person to be their own pre-disease control, reducing the genetic variation that is inherent in population based studies. Based on these results, the minimal damage to the integrity of the plasma proteome with long-term storage does not outweigh the benefits that maybe generated by using these samples.

## Participating CHS Investigators and Institutions

(*All Investigators here listed have provided signed permission to be acknowledged*)

Steering Committee Chairman: Curt D. Furberg, MD PhD, Wake Forest University School of Medicine; NHLBI Project Office: Jean Olson, MD MPH; Wake Forest University School of Medicine: Gregory L. Burke, MD; Wake Forest University — ECG Reading Center: Pentti M. Rautaharju, MD PhD; University of California, Davis: John Robbins, MD MHS; The Johns Hopkins University: Linda P. Fried, MD MPH; The Johns Hopkins University — MRI Reading Center: Nick Bryan, MD PhD; Norman J. Beauchamp, MD; University of Pittsburgh: Lewis H. Kuller, MD DrPH; UniversityofCalifornia, Irvine—Echocardiography Reading Center (baseline): Julius M. Gardin, MD; Georgetown Medical Center — Echocardiography Reading Center (follow-up): John S. Gottdiener, MD; New England Medical Center, Boston — Ultrasound Reading Center: Daniel H. O’Leary, MD; University of Vermont — Central Blood Analysis Laboratory: Russell P. Tracy, PhD; University of Arizona, Tucson — Pulmonary Reading Center: Paul Enright, MD; Retinal Reading Center — University of Wisconsin: Ronald Klein, MD; University of Washington — Coordinating Center: Richard A. Kronmal, PhD.

## Figures and Tables

**Table 1:** Characteristics of the study population

Characteristic	N	%
Age (years)	Mean=74.4	SD=3.4
Gender		
Male	17	34.0
Female	33	66.0
Race		
White	41	82.0
Black	9	18.0
Clinical Site		
Wake Forest University	18	36.0
University CA, Davis	10	20.0
Johns Hopkins	10	20.0
University of Pittsburgh	12	24.0

Abbreviation: SD = Standard Deviation.

**Table 2:** Changes in peak intensity following freeze-thaw cycle

	Changes in peak intensity				
Number of freeze/thaw cycles	25^th^ percentile	Median	75^th^ percentile	95^th^ percentile	99^th^ percentile
2	0.7%	1.7%	3.0%	8.3%	19.1%
3	1.0%	2.4%	4.6%	13.8%	43.0%
4	1.3%	3.5%	7.5%	27.4%	67.6%
5	1.2%	3.1%	7.0%	25.8%	51.7%

**Table 3:** Changes in peak intensity following freeze-thaw cycles by mass/charge

Number of freeze/thaw cycles	Median changes in peak intensity			
	35,000	<5,000–10,000	10,000–20,000	≥20,000
2	0.242%	0.687%	0.197%	−0.306%
3	−0.344%	3.419%	−0.621%	−0.508%
4	−0.330%	3.218%	−0.099%	−0.295%
5	−1.201%	2.018%	−0.918%	−0.587%

**Table 4:** Peak number, variation and correlation of plasma samples from 1992/93 compared to 1996/97

	1992/93 (25^th^, 75^th^ percentile)	1996/97 (25^th^, 75^th^ percentile)
Number of peaks	636 (611, 666)	630 (596, 673)
Coefficient of Variation	14.6% (11.6%, 26.3%)	13.7% (11.3%, 26.5%)
Coefficient of Variation using 5 replicates	11.1% (8.0%, 18.8%)	10.2% (7.9%, 19.6%)
Correlation	0.40 (0.32, 0.47)	

**Table 5:** Comparison of the number of peaks at different mass/charge ranges in the 1992/93 and 1996/97 samples

Mass/charge range	Median number of peaks (25^th^, 75^th^ percentile)	
	1992/93	1996/97
< 5,000	174 (163,191)	177 (163,194)
5,000 – 10,000	58 (53,65)	57 (53,63)
>10,000	401 (386,426)	395 (376,421)
